# Supplementary material for: Computational multiplex panel reduction to maximize information retention in breast cancer tissue microarrays
Source: PLoS Comput Biol. 2022 Sep 30;18(9):e1010505. doi: 10.1371/journal.pcbi.1010505 (PMC9555662; doi:10.1371/journal.pcbi.1010505)
Supplement: S1 Fig — The average SSIM was measured for each stain individually and averaged. Likewise, the Spearman correlation between the original stain intensity and the resultant stain intensity was calculated for each stain independently and averaged across the withheld panel set. (PDF) [file pcbi.1010505.s001.pdf]

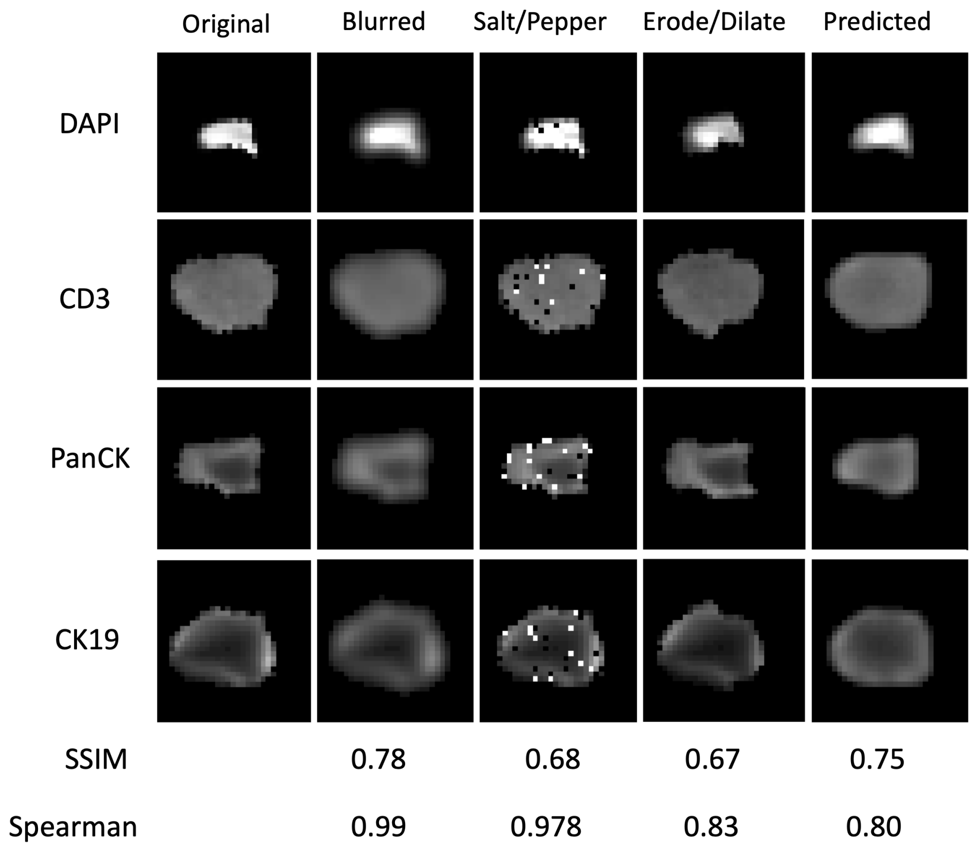

Figure S1. To frame the extent of error in the predicted results from a randomly selected reduced panel of 12 stains, several technical noises were simulated and evaluated for the same metrics. The average SSIM was measured for each stain individually and averaged. Likewise, the Spearman correlation between the original stain intensity and the resultant stain intensity was calculated for each stain independently and averaged across the withheld panel set.
