# Supplementary material for: Computational multiplex panel reduction to maximize information retention in breast cancer tissue microarrays
Source: PLoS Comput Biol. 2022 Sep 30;18(9):e1010505. doi: 10.1371/journal.pcbi.1010505 (PMC9555662; doi:10.1371/journal.pcbi.1010505)
Supplement: Attachment — Submitted filename: ternes_staining_review.pdf [file pcbi.1010505.s008.pdf]

Ternes et al. evaluate four different methods for reducing marker panel size in a multi-plex imaging experiment. They compare performance of each panel reduction method by applying image-to-image translation and evaluating how well the reduced marker set reconstructs the full marker set. Specifically, they apply their previously developed ME-VAE algorithm to perform the translation. They evaluate performance in three different ways: 1) mean intensity correlation of original and reconstructed images, 2) structural similarity index measure, and 3) cell type cluster matching via normalized mutual information. The authors also assess generalizability by applying their highest scoring marker selection panel to a separate TMA dataset.

Overall, the paper presents an interesting approach to solve an important and timely biological problem, and the existing experiments are well controlled. However, the extent to which the conclusions are generalizable and exactly how a reader would benefit from their findings is unclear. I think the paper would dramatically improve with substantially more methodological detail, increased clarity of usage and limitations, and by extending the model generalizability assessment. I outline several concerns in more detail below:

- My primary concern is that we don't know if the marker reduction algorithm selection is specific to only CyCIF staining in tissue microarrays as identified by only one specific image-to-image translation method. In other words, a reader might be misled in choosing a reduced marker panel if the ME-VAE image-to-image translation fails to generalize to their experimental design. Would other image-to-image translation models select the same markers? Does this work with other staining and data modalities? Would the approach work in diseases other than cancer? The generalizability section assesses performance of a held-out dataset, which is great, but the data are also cancer TMAs. Currently, the methods and results fall short of the all-encompassing conclusions and title.

Other concerns:

- The authors should clarify early in the paper that the reduced panel marker selection algorithms cannot be used in new panels. They can only be applied after initial measurements. This is ok (what else are pilot datasets for?) but this needs to be clear in the introduction.
- The authors should provide examples of markers in the introduction – are the authors talking about targeted markers? Does marker specificity matter? What about general markers that stain for cell structures?
- The authors should provide much more method details. Individually, these all are easily fixed; taken together, they provide serious reproducibility concerns.
    - What is Structural Similarity Index Measure (SSIM) – no citations or explanations are provided. This evaluation method is cited in the results with slightly more detail, but the methods should contain a citation, much more information about the method and implementation, as well as an intuitive interpretation.
    - How does NMI evaluate retention of information necessary for downstream phenotyping? What downstream phenotyping did the authors perform? I believe

the authors are referring to a cell type cluster match analysis, but isn't this analysis sensitive to clustering algorithm? How does the clustering algorithm influence cluster matching?

    - What metrics did the authors use to measure and determine elbow for k-means?
    - The authors should provide citations and versions for all software used (e.g. UMAP)
    - The authors should provide more details for simulating technical noise
        - What does mode "s&p" do?
        - There is a mixture of "I" and "we" in this section
        - For erosion and dilation, how did the authors choose which half of the image?
        - Add citation to supplementary figure 1 in this section – it is helpful!
    - The authors should provide much more details on their deep learning gradient-based selection method. How are the authors aggregating the gradient to calculate channel importance? Is it simply adding weights?
    - More details on the markers are required
        - What structures are they marking?
        - What vendors did the authors obtain them from?
    - The authors should make their analysis code publicly available.
        - The authors present https://github.com/GelatinFrogs/ME-VAE_Architecture, but this repository does not contain the analysis code used in this paper from what I could tell.
    - The authors should provide their supplementary tables in a parsable format
- The authors make an important point that their marker prediction is often no worse than random noise (Figure S1). This is an important finding that I mis-interpreted on my first read-through, and one I think can be better explained.
    - Comparing reconstruction of the 12 stains against the noise analysis, it is unclear what the authors did. The authors calculate SSIM in real vs. blurred, and real vs. salt/pepper, etc? Or, do the authors introduce noise prior to training and then compare SSIM?
- "This baseline of 1-to-1 substitution resulted in subpar predictions that did not converge to a correlation approximating the full panel until nearly all stains were included in the reduced set, indicating the need for predictive models to retain information on removed markers."
    - Missing reference to figure 3
- Figure 3 describes mean performance, but don't we also care about variance? A reader might care about predicting all stains reasonably well, than predicting some stains extremely well and others not at all.
- The authors might consider adding to Figure 2 some compilation of multiple stains to visualize complexity the full-panel imputation
- It would be great to see predictions on multiple random 50% panel selection. What is the range of SSIM and Spearman correlations? Did the authors just get lucky with the 50% selected?

- What are the x and y axes in figure 4 and 5?
- The authors might consider adding a table describing performance for all evaluations

Gregory P. Way, PhD
University of Colorado Anschutz
