# Supplementary material for: Computational multiplex panel reduction to maximize information retention in breast cancer tissue microarrays
Source: PLoS Comput Biol. 2022 Sep 30;18(9):e1010505. doi: 10.1371/journal.pcbi.1010505 (PMC9555662; doi:10.1371/journal.pcbi.1010505)
Supplement: Attachment — Submitted filename: PanelReductionPaper-IndividualResponses-Final.pdf [file pcbi.1010505.s009.pdf]

Dear Reviewers and Editors,

On behalf of the authors, I wish to extend our sincere thanks for your time and effort focused on improving our work. All comments from each of the reviewers are presented below along with our response indicating how we incorporated feedback into our manuscript. Please find attached an updated manuscript with revised changes which have all been tracked.

**Title**: Computational Multiplex Panel Reduction to Maximize Information Retention
(revised) Computational Multiplex Panel Reduction to Maximize Information Retention in Breast Cancer Tissue Microarrays

**Authors**: Luke Ternes, Joe W. Gray, and Young Hwan Chang

**Comments from Reviewer #1**

Summary

The authors propose to address a very interesting and impactful problem, of how to select a reduced set of stains in multiplexed images, that capture a similar amount of information as a larger panel. Their main claim is that, out of several strategies, using a simple metric of correlation between stains to determine which stains to withhold performs well and generalizes to unseen cell lines within breast cancer cells. However, I think there is one fatal flaw in the experimenting supporting this claim: because each of the withholding strategies results in different stains being predicted by their models, and the metrics are reported on reconstructing the particular stains withheld from a model, the metrics between models in this paper are not comparable. This is because different stains can be more or less difficult to predict: more spatially variable stains are more challenging than homogeneously distributed stains, and from my reading, there has been no attempt to fairly compare models where the more challenging stains are given as training inputs versus being held out as test inputs.

A second major flaw is that the authors claim that they can choose stains that maximize the "information" retained in this paper, but as far as I can tell, the evidence supporting this is weak. The only attempt at showing that biologically relevant information is retained is Figure 6, which shows that clustering mean intensity (I think, the writing is somewhat ambiguous) from a reduced set of stains generally has around 0.60 normalized mutual information with the full panel of stains. However, this experiment is weak for multiple reasons: there is no evidence that clustering of single cell features in this context retrieves cell types; as far as I can tell, there is only a very limited attempt to extract features from the images (i.e. only intensity, not shape or distribution); the clustering method and parameterization is arbitrary and simple, and there appear to be subtypes of cells in the dimensionality reduction that the clustering method is not retrieving in the full panel if we even believe this metric biologically relevant; and there is no strong baseline to interpret what a 0.60 NMI even means in this context (only a very weak randomly shuffled baseline). Otherwise, the rest of the paper shows evidence that the distribution of stains can be predicted well in and out of training distribution, which is not the same as the titular claim.

There are other comments I have about the clarity of the paper, some technical points of the methodology, and the metrics used - I hope these will be helpful for the authors. But given that the experiments supporting the main claims of the paper are technically flawed, and only weakly support the titular claim of the paper at best, I have no choice but to recommend a reject unless the authors can

demonstrate to me that I've missed something major in their work.

Major Comments

Comment 1.1:

- The primary metric supporting most of the claims in this paper is the reconstruction quality of unseen stains, measured by the Spearman correlation and SSIM between the predicted outputs for these stains and their actual stains. This is used to support the claim that a simple metric of marker correlation performs best at selecting a maximally informative reduced subset of markers. I would like to posit an alternative hypothesis: the marker correlation metric is selecting stains to withhold that are most spatially homogeneous (i.e. more uniformly distributed across the cell/nucleus). These stains are systematically easier to predict, so the reason why we're seeing higher correlations is simply because some models have been given harder images to predict (e.g. more spatially heterogeneous images), while others have been given easier ones. I would like to see this ruled out by showing a baseline where the N channels being predicted are the most spatially homogeneous channels. More broadly, I would like to see the issue that some models are given easier prediction problems than others be addressed - the metrics across models are just not comparable currently. One solution would be to have a single standard set of held out markers, and then choose the subset along the remaining markers that best predicts these markers, instead of reporting a metric on variable outputs.

Response 1.1:

- One important point of note here is that the prediction architecture that reconstructs the full panel images is kept consistent throughout tests. The selection method is performed independently from the full panel prediction. (See Figure 1 and Methods section on imputation architecture). The reviewer is correct that some selection methods might be selecting markers in such a way that the withheld stains are difficult to predict. Selection methods that only select easy to reconstruct stains will perform poorly in evaluation, which is what we would want to see. If a selection method is always going to select easy to reconstruct stains for the reduced panel, thus making withheld panel reconstruction difficult, then those selection methods should not be used. In this experiment we are not testing reconstruction architectures, only selection methods. If we were testing reconstruction architectures, we would want to use standard set inputs for comparable metrics as reviewer suggested, but that is not what we are focusing on. Here we are evaluating panel selection methods. Each selection method is given the same full panel information to select from, and then we evaluate its selection using the same reconstruction architecture across all selection methods for comparable evaluation of reduced panels. Furthermore, as shown in Figures 3, 4, and 5, methods were evaluated using both the withheld markers specific to each selection method, but also the full panel, which is shared across all methods. Thus, we believe that the metrics between selection models are comparable.
- Reviewer suggested to use a standard set of held out markers but it does not help to evaluate the methods for selection a sub-panel. There are three main flaws to this suggestion. First, this method does not guarantee fair evaluation. One method might perform well at reconstructing the withheld/not-evaluated makers but perform poorly on the withheld/evaluated markers. And another method might perform oppositely. Evaluation in such a manner would skew analysis and result in a fatal flaw in reduced panel selection. Second, the panel selection methods will be limited by not being able to select the withheld markers, which might be necessary for creating

the ideal reduced panel. By removing them from the selection process, you are withholding key information that is necessary for selection and reconstruction. Also, selecting the withheld markers will be arbitrary. Third is that the very goal of these methods is to select a sub-panel that makes reconstruction easy. If some selection methods are prone to selecting sparse stains, then it is only fair that they be penalized by being evaluated based on the difficult to reconstruct stains they chose to withhold for themselves. By giving all methods the same standard evaluation set, we are no longer evaluating the full context of the models' marker selections.

Comment 1.2:

- Is this paper about single cell phenotyping, or single nuclei? The segmentation method used seems to work on nuclear dyes exclusively, which suggests the latter, but the paper seems to suggest the former. This distinction is important and would impact the biological claims and limitations of the paper: reconstructing purely nuclear expression/localization is easier than reconstructing expression at the full cell level (which I expect would be much more difficult due to increased number of compartments and variability in cell morphology). It is not clear that a method built for imputation of stains in the nuclei exclusively would extend to full cells in general: the authors need to clarify this and better contextualize their method.

Response 1.2:

- The cell segmentations used here were obtained using a nuclear segmentation model, then the masks were dilated to obtain cell masks. Cell masks were generated by UnMicst/S3seg using MCMICRO pipeline where the cell ring mask were dilated 3 pixels from nuclear masks. This has been updated to be clearer in the Methods. Although initial segmentations were obtained from the using the nuclei, Figure 2 demonstrates that the dilated cell masks capture expression differences and localization within the cellular morphology.

Comment 1.3:

- I'm confused about the evaluation in Figure 6, which seems to be very critically important given that this is the only experiment that attempts to validate whether the predicted stains have any utility to downstream biological analyses and not are just technically correct reconstructions of the stains. How are the authors obtaining the latent space for the full panel - is this just the average intensity of the cell per stain or do the authors have some kind of latent space for the full panel intensities? If it's the former, how does this relate to point 4? How was k for k-means selected? There's a lot of substructure in the full panel intensities (e.g. especially in Cluster 7) that doesn't seem to be resolved by any of the subsampled models - doesn't this suggest that there is information in the full panel being missed by the partial panels? In general, the arbitrary selection of clustering method and co-efficient, the limited features being employed here, and the lack of solid baselines for interpreting the NMI means that the claim that the deep learning architectures are capturing the "most defining" information of a full panel is fuzzy.

Response 1.3

- As stated in the caption of Figure 6 and in the Methods, the clustering was performed on the intensities of the full panel reconstructions, and the reviewer is correct, it is just the average intensity of stain per cell. The clustering was not performed on image latent spaces. While the segmentation, as mentioned in Comment 1.4, would affect the mean intensities extract, the cell segmentations here do not impact this evaluation. The measurements are taken on a single cell level, so any artifacts from segmentation are reflected both in the full panel and in the reconstructed panels. This methodology shown here is not attempting to remove segmentation

artifact or improve erroneous data, simply to re-capture information from a reduced set and show that it is useful for downstream analysis. Additionally, since cell segmentations are used, there is localization and morphology information, however, those are assessed with SSIM (Figure 2 and corresponding text), and Figure 6 only assesses the use of reconstructed mean intensities for downstream clustering.

- As stated in the caption of Figure 6, a k=10 for k-means was selected using the elbow method for the silhouette score, as is a common practice. We have made this more clear in the Methods.
- As the reviewer points out there are some cluster sub features that are being missed between the full panel and the reconstructions, and this is true across all selection methods, including the highest performing correlation-based method. We attempt not to oversell the capabilities of the reconstructions in this paper, stating that an intelligent selection method does not show significant improvements from random selection when producing clusters that match the original full panel. We do still hold that the most defining information was reconstructed adequately with the selection methods as they are able to achieve macro-level similarity in clusters, even if some of the more nuanced information is not retained. We do not try to claim that all information is retained, just that we can maximize the amount of information via panel selection and reconstruction.

Comment 1.4:

- The segmentation procedure would interact with the experiments reported in this paper along multiple axes, but the paper doesn't go in enough detail about how exactly this was done. Greater clarity on the methods here would be appreciated. First, are stains used for segmentation also predicted outputs of the ME-VAE in various stratification set-ups? If so, how do the authors rule out that the stains are being predicted based upon the other stains, and not simply due to the segmentation mask? It seems that the segmentation mask would contain information about the localization of the stains, and potentially their intensity. Any stains used for segmentation should be excluded from the evaluation of the model. Second, how prone is this model to artifacts especially on this new, unseen data? My concern is that if the model is identifying artifacts or background as nuclei/cells, these kinds of mis-segmentations would tend to be bright or dim along all channels, so these would be easier to get.

Response 1.4:

- Stains used for segmentation were included in the selection and panels for reconstruction. We address the above concerns, not by removing them from the panel, but by forcing them to be included, as stated with Hoechst. Since it is a part of the segmentation method, we concluded that it is necessary to include the segmentation stains in any reduced panel for the dataset. All selection methods used the same segmentations, so there is no unfair advantage from one method to another. Additionally, it is most appropriate to use the segmentation stains in the reconstruction if they improve the information retained. For instance, Hoechst visualizes nuclear DNA, and it has been used to assess cell morphology or cell cycle phase estimation, thus it is a potentially important marker to predict other marker expression in the reduced panel setting. When conducting a real-world experiment, a researcher will not want to exclude information that can help in retaining information and imputing a full dataset, regardless of whether that information comes from the segmentation mask or the reduced panel intensities.

Comment 1.5:

- As a practical point, do the authors consider spatial structure or intensity of stain to be the critical aspect to predict in these problems? My view is that the Spearman correlation is only going to capture the former, not the latter. But especially if this is nuclear expression, it seems like intensity is important for profiling here. Many of these stains appear to be transcription factors, where concentration in the nuclei is going to be important and not just spatial distribution. How well do the metrics reported in this paper align with the downstream profiling aims, given that it's possible to get the intensity of the marker totally wrong but still get a high correlation as long as the ranking of the pixels is preserved? (Also, is histogram stretching done per image, or globally using the percentile across all images? This seems critical as well.)

Response 1.5:
- As described in the Methods, Spearman correlation was conducted on the mean intensities per single cell for each stain, not tissue level. From our reading, we believe that some of this reviewer's comments are based on tissue level comparison, for instance, the reviewer mentioned "spatially variable stains" or "homogeneously distributed stains" in earlier comments.
- We correlate the mean intensities of the full panel to the reconstructed panels, with each single cell being a data point. When we evaluate the models using the Spearman correlation, we are evaluating the intensity information, not the spatial structure as the reviewer suggests.
- We consider both the intensity and the structure of the staining pattern in single cell image to be important. This is why we evaluated the reconstructions with both Spearman correlation and SSIM (structural similarity index). While the reviewer is correct that correlation will not account for concentration of stain in different portions of the image, SSIM will account for the location and intensity throughout the regions of the cell to give a more robust measure of the image.
- Histogram stretching was done globally across all TMAs, and each stain was stretched independently.

**Comments from Reviewer #2**

Summary

The authors develop a VAE to predict certain channels of multiplexed images from other channels. They apply several selection algorithms to identify the most predictive set of markers, and the set of markers than can be predicted. Their work can be used to guide panel design, which is a very important aspect in experiment design. The pipeline requires full panel data to identify the set of most predictive markers, suggesting the need for preliminary studies for each experiment, or use of prior knowledge. The manuscript establishes the feasibility of predicting certain markers from others, and establishes a data driven approach for marker selection. Future studies building on the current work can benefit panel design in the long run. Although I find the paper very interesting, there are a few concerns the authors need to address.

Major Comments

Comment 2.1:
- Given the study is done on one dataset, it is not clear if the correlations between true mean intensities and predictions are due to the power of deep learning models in extracting marker relations, or an artifact of panel design measuring heavily correlated markers in the first place. I would appreciate if the authors could comment if other multiplexed datasets show similar

overall marker correlations, and if they believe similar correlations can generally be found in other datasets.

Response 2.1:

- Below is an example figure showing the similar pattern of panel correlations across for staining intensities. This data was obtained on a different multiplexed tissue imaging platform (MACSima Imaging System – Miltenyi Biotec, not shown in the current manuscript) with a different panel and biological sample from those used in our paper.

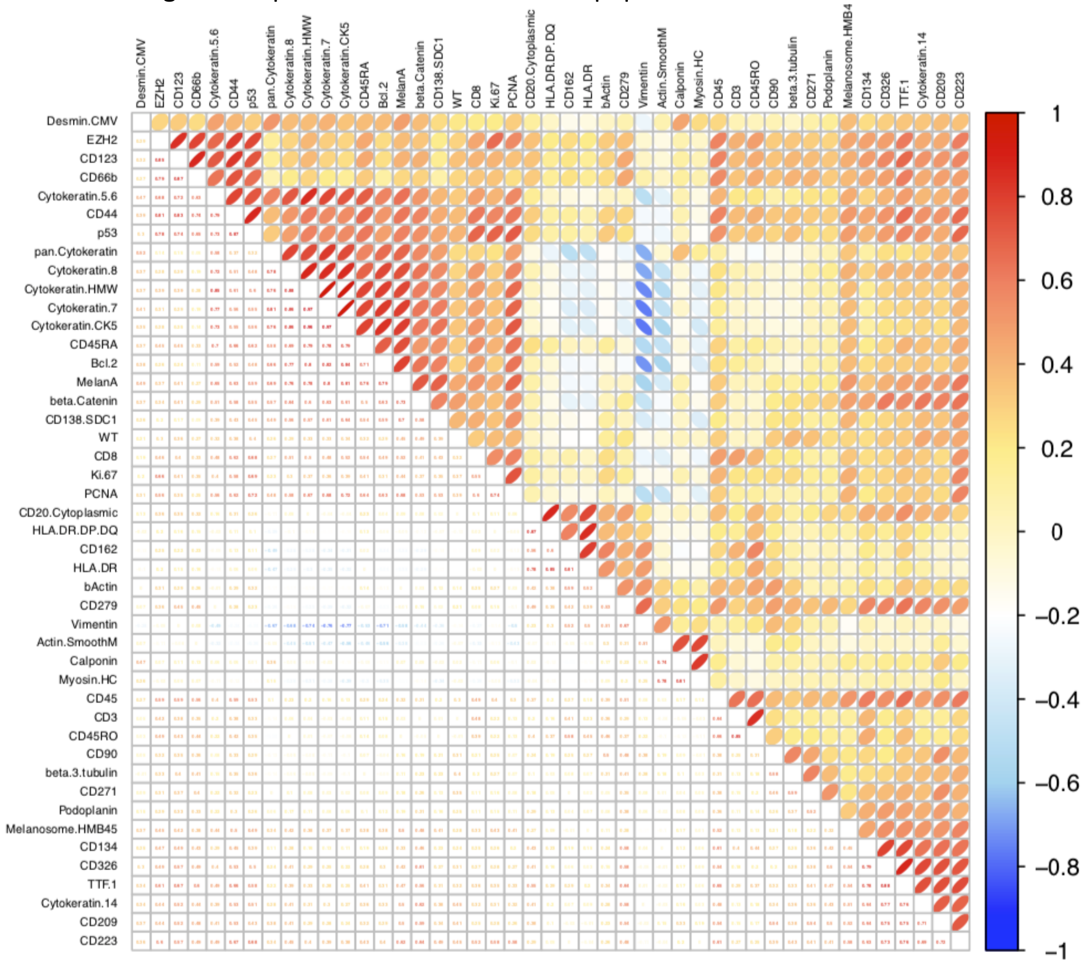


- While it is true that the power of deep learning plays a large role in being able to extract marker relations, we can see that the selection of a reduced panel does impact how well the markers are reconstructed. Correlation-based selection plays into the marker correlations present on the panel by selecting a reduced set for which the model can easily extract and reconstruct these relations. The other methods do not guarantee that the paired information will be present and therefore the model struggles to reconstruct information that is not present in the reduced panel. As can be seen in the attached figure, many multiplex panels have a large overlap of correlating markers, which will naturally happen as the number of stains increases. If, however, there were to be a multiplex experiment for which the full panel had no/few correlating markers, then correlation-based selection might not perform the best since there are no significant correlations to select based on.

Comment 2.2:

- The authors need to better explain the train/test split in their methods section, e.g., if the train/test split is done at the cell level, image level, or patient level. The authors need to study how much the mean intensity prediction correlations may vary across patients, and how patient specific artifacts may affect the predictions of their model.

Response 2.2:

- Selection methods were run using the correlations and interactions across the full dataset. The image reconstruction model was trained using a random 90/10 test split performed at the cellular image level. The test set was then used for comparative analysis of the selection methods, i.e. reconstruction accuracy, correlation analysis, and clustering. This has been updated in the Methods section of the manuscript.
- While we agree that model performance and panel selection will vary from patient to patient, we address this in the manuscript, stating that models may not perform well when unseen correlation and biological features are present in new/unseen patients or cancer subtypes. Here we tested generalizability across several breast cancer subtypes. We do not see the need to test the same trend across patients as we have already addressed it as a limitation in the manuscript.

Comment 2.3:

- Sparse subspace-based panel selection method performs inferior to the simpler correlation-based panel selection method. I was wondering if the authors could comment why the subspace model performs inferior given that its assumptions on marker relations better conform to data properties.

Response 2.3:

- We agree with the reviewer, and it was our initial hypothesis that the subspace model would perform the best out of the tested methods. This was not the case however. We believe that the problem with the subspace selection method stems from the method by which the markers are selected after computing the resultant interaction map. The interaction map shows how a stain intensity can be reconstructed from multiple other stains in combination, but does not intuitively say which stains should be used for a reduced panel. We tried several methods for using the resultant interaction map for creating a reduced panel (only the best performing method shown and discussed in the paper). This included clustering the interaction map, such that similarly predicted stains were together and clustering such that similarly predictive stains were together. The selection method that worked the best, however, was computing a panel score by combinatorially testing reduced panels to maximize interactions between the reduced panel and the withheld panel and minimizing interactions within the reduced panel. While we also expect this method to have performed better, it is likely due to not finding an adequate panel selection method that used the interaction map.

Comment 2.4:

- I was wondering if the authors could explain their choice of cost function for subspace-based panel selection. The authors use the L2 norm of error ( $|| I-CI ||_2$) instead of the conventional squared L2 norm ($|| I-CI ||^2_2$), and use L2 penalization followed by thresholding instead of a small L1 or elastic-net penalty to get a sparse solution.

Response 2.4:

- The choice of loss is based on existing implementations of subspace-based models: https://github.com/panji530/Deep-subspace-clustering-networks
- The loss we used here is in fact the squared L2 norm that is suggested by the reviewer. This is an annotation error on our part. We have corrected is in the revised manuscript.

Comment 2.5:

- The authors need to do more benchmarking of their VAE. Since the final mean intensity is the main objective, the authors need to compare their VAE with models that directly predict mean intensities. Despite the pixel level VAE can take advantage of subcellular spatial relations among markers, the extent the added complexity enhances prediction accuracy is not clear. This is important since in the marker selection step correlation-based selection outperformed more complex algorithms.

Response 2.5:

- Although we use the final reconstructed mean intensities for several metrics, we also use SSIM to evaluate the actual structure and localization of the stains within the reconstructed images. Our goal is to maximize the amount of information that is retained (including morpho-spatial features) which go beyond simple mean intensity. A pixel level VAE is necessary for capturing such features. Moreover, although there will most likely be better image-to-image translation architectures than the one used here (and better intensity to intensity architectures), the main goal of this paper was to evaluate selection methods for panel design and demonstrate the capacity for a reconstructive model to maximize the amount of information retained using a reduced panel.

Comment 2.6:

- I would appreciate if the authors could comment if they believe panel reduction generalizes to other multiplexed imaging techniques, such as IMC.

Response 2.6:

- The authors believe that the principles behind image reconstruction and the embedding of image features across multiple channels is applicable to many multiplex platforms including CyCIF and CODEX, and prior research supports this (see citation 7 and 11). Image translation has even been used to find common features in H&E and fluorescent images to enable stain prediction. Likewise we believe that the idea of sub-panels containing relevant information regarding a full panel is applicable to other imaging modalities as well. Within this experiment we show that it works with CyCIF, and although we hypothesize that it will extend, we have not tested that here.

**Comments from Reviewer #3**

<u>Summary</u>

Ternes et al. evaluate four different methods for reducing marker panel size in a multi-plex imaging experiment. They compare performance of each panel reduction method by applying image-to-image translation and evaluating how well the reduced marker set reconstructs the full marker set. Specifically, they apply their previously developed ME-VAE algorithm to perform the translation. They evaluate performance in three different ways: 1) mean intensity correlation of original and reconstructed images, 2) structural similarity index measure, and 3) cell type cluster matching via normalized mutual

information. The authors also assess generalizability by applying their highest scoring marker selection panel to a separate TMA dataset.

Overall, the paper presents an interesting approach to solve an important and timely biological problem, and the existing experiments are well controlled. However, the extent to which the conclusions are generalizable and exactly how a reader would benefit from their findings is unclear. I think the paper would dramatically improve with substantially more methodological detail, increased clarity of usage and limitations, and by extending the model generalizability assessment. I outline several concerns in more detail below:

• My primary concern is that we don't know if the marker reduction algorithm selection is specific to only CyCIF staining in tissue microarrays as identified by only one specific image-to-image translation method. In other words, a reader might be misled in choosing a reduced marker panel if the ME-VAE image-to-image translation fails to generalize to their experimental design. Would other image-to-image translation models select the same markers? Does this work with other staining and data modalities? Would the approach work in diseases other than cancer? The generalizability section assesses performance of a held-out dataset, which is great, but the data are also cancer TMAs. Currently, the methods and results fall short of the all-encompassing conclusions and title.

Response:

- These TMAs represent numerous patients and several different breast cancer sub-types (Luminal A, Luminal B, Luminal B/HER2, HER2, TN, ILC, Normal Breast). For that reason, although the dataset is all TMA data, we would argue that the dataset is heterogeneous. It is possible that some datasets and panels might not be as well predicted using the methods described (i.e, a panel where there is no/minimal correlation between stains), but it is not possible to make claims for all such scenarios. Here we simply test several methods for reduced panel selection and show that they can be used for retaining information about the full panel using image reconstruction. It is possible that different image-to-image translation algorithms will perform better with different sub-panels and therefore perform better with different selection methods. Again, we are not attempting to propose a single algorithm to be used in all panel reduction tasks and experiment settings. We are providing a proof-of-concept for intelligent selection and an evaluation of several (not all-encompassing) methods to do so and we believe that users of multiplex imaging techniques would benefit from this ability to save room for the other markers if the reduced marker panel can predict all panel information. We have updated our title and manuscript to indicate a more targeted scope of the experiment to breast cancer TMAs imaged with CyCIF.

<u>Major Comments</u>

Comment 3.1:

- The authors should clarify early in the paper that the reduced panel marker selection algorithms cannot be used in new panels. They can only be applied after initial measurements. This is ok (what else are pilot datasets for?) but this needs to be clear in the introduction.

Response 3.1:

- This clarification has been included in the introduction of the paper.

Comment 3.2:

- The authors should provide examples of markers in the introduction – are the authors talking about targeted markers? Does marker specificity matter? What about general markers that stain for cell structures?

Response 3.2:

- Within the main text, we have included a brief explanation of a few of the markers in the panel that cover a range of marker types and purposes.

Comment 3.3:

- The authors should provide much more method details. Individually, these all are easily fixed; taken together, they provide serious reproducibility concerns.

Comment 3.3a:

  - What is Structural Similarity Index Measure (SSIM) – no citations or explanations are provided. This evaluation method is cited in the results with slightly more detail, but the methods should contain a citation, much more information about the method and implementation, as well as an intuitive interpretation.

Response 3.3a

- We have included a brief explanation of what SSIM is, why it was chosen, and given a citation for it in the Methods section.

Comment 3.3b:

  - How does NMI evaluate retention of information necessary for downstream phenotyping? What downstream phenotyping did the authors perform? I believe the authors are referring to a cell type cluster match analysis, but isn't this analysis sensitive to clustering algorithm? How does the clustering algorithm influence cluster matching?

Response 3.3b:

- We have added in an additional standard clustering method, PhenoGraph, to our analysis. NMI, which is a calculation of mutual information between clusters, shows similar scores to those predicted with k-means, indicating that the analysis is consistent across clustering methods.

Comment 3.3c:

  - What metrics did the authors use to measure and determine elbow for k-means?

Response 3.3c:

- As stated in the caption of Figure 6, silhouette score was used to determine the elbow. This has been further clarified in the Methods section.

Comment 3.3d:

- The authors should provide citations and versions for all software used (e.g. UMAP)

Response 3.3d:

- We have updated citations for all our software packages.

Comment 3.3e:

- The authors should provide more details for simulating technical noise
  - What does mode “s&p” do?
  - There is a mixture of “I” and “we” in this section
  - For erosion and dilation, how did the authors choose which half of the image?
  - Add citation to supplementary figure 1 in this section – it is helpful!

Response 3.3e:

- “s&p” stands for “salt and pepper” which is a form of random image noise where pixels are activated and deactivated. This has been updated in the manuscript.
- Erosion and dilation halves were arbitrarily chosen and kept consistent throughout the dataset.
- Typographical and citations have been updated.

Comment 3.3f:

- The authors should provide much more details on their deep learning gradientbased selection method. How are the authors aggregating the gradient to calculate channel importance? Is it simply adding weights?

Response 3.3f:

- Using the same method as described in the cited reference, the tensorflow built-in method of GradientTape was used to track the gradient at the embedding layer. This explanation has been added to the manuscript.

Comment 3.3g:

- More details on the markers are required
  - What structures are they marking?
  - What vendors did the authors obtain them from?

Response 3.3g:

- The Vendor and catalog number information necessary to reproduce the staining and obtain information regarding markers has been added to the supplemental figure.

Comment 3.3h:

- The authors should make their analysis code publicly available.

- The authors present https://github.com/GelatinFrogs/MEVAE_Architecture, but this repository does not contain the analysis code used in this paper from what I could tell.

Response 3.3h:
- We have added and linked code for the three selection methods used in our analysis.

Comment 3.3I:
- The authors should provide their supplementary tables in a parsable format

Response 3.3I:
- The supplementary figures have been included as .csv files.

Comment 3.4:
- The authors make an important point that their marker prediction is often no worse than random noise (Figure S1). This is an important finding that I mis-interpreted on my first read-through, and one I think can be better explained.
  - Comparing reconstruction of the 12 stains against the noise analysis, it is unclear what the authors did. The authors calculate SSIM in real vs. blurred, and real vs. salt/pepper, etc? Or, do the authors introduce noise prior to training and then compare SSIM?

Response 3.4:
- When we state that it is within the margins of normal technical variation, we are stating that the noise from artifacts of blurring, speckling, and segmentation have a similar SSIM and correlation as stain imputation. This means that the reconstructions perform well and are within the realms of artifact variation that are normally accepted as a part of imaging. Further clarification our intended meaning has been added to the manuscript.

Comment 3.5:
- "This baseline of 1-to-1 substitution resulted in subpar predictions that did not converge to a correlation approximating the full panel until nearly all stains were included in the reduced set, indicating the need for predictive models to retain information on removed markers."
  - Missing reference to figure 3

Response 3.5:
- The reference has been included in the manuscript.

Comment 3.6:

- Figure 3 describes mean performance, but don't we also care about variance? A reader might care about predicting all stains reasonably well, than predicting some stains extremely well and others not at all.

Response 3.6:
- Correlation variance has been calculated as requested. The values have been added to the text of the results and to the figure mentioned.

Comment 3.7:
- The authors might consider adding to Figure 2 some compilation of multiple stains to visualize complexity the full-panel imputation

Response 3.7:
- An additional column has been added to the figure to show the compilation of the stains for both the training set and the withheld set of markers.

Comment 3.8:
- It would be great to see predictions on multiple random 50% panel selection. What is the range of SSIM and Spearman correlations? Did the authors just get lucky with the 50% selected?

Response 3.8:
- Although repeating multiple instances of random sampling at 50% would strengthen the ground that random selection can provide adequate information for reconstruction, the purpose of this paper is to determine and suggest an intelligent method for panel selection, and our results already show that other intelligent selection methods perform well. Re-showing that random selection performs well does not strengthen this argument. And the reviewer is likely correct that there is a random sub-sample of stains that would perform very poorly. The use of the random proof-of-concept here was simply to show that reconstruction can be done with a reduced panel set.

Comment 3.9:
- What are the x and y axes in figure 4 and 5?

Response 3.9:
- These figures have been updated.

Comment 3.10:
- The authors might consider adding a table describing performance for all evaluations

Response 3.10:
- We have added a table to the end for direct comparison of all methods for the metrics we calculated.
