# Supplementary material for: Computational multiplex panel reduction to maximize information retention in breast cancer tissue microarrays
Source: PLoS Comput Biol. 2022 Sep 30;18(9):e1010505. doi: 10.1371/journal.pcbi.1010505 (PMC9555662; doi:10.1371/journal.pcbi.1010505)
Supplement: Attachment — Submitted filename: PanelReductionPaper-SecondRebutal-IndividualResponses_Final.docx [file pcbi.1010505.s010.docx]

Dear Reviewers and Editors,

On behalf of the authors, I wish to extend our sincere thanks for your time and effort focused on improving our work. All comments from each of the reviewers are presented below along with our response indicating how we incorporated feedback into our manuscript. Please find attached an updated manuscript with revised changes which have all been tracked.

**Title**: Computational Multiplex Panel Reduction to Maximize Information Retention

**Authors**: Luke Ternes, Jia-Ren Lin, Yu-An Chen, Joe W. Gray, Peter K. Sorger and Young Hwan Chang

**Comments from Reviewer #1**

Summary

Overall, the authors' comments have improved the clarity of their work: I now better appreciate that the authors trying to describe a method to select a reduced subset of stains agnostic of method, rather than reporting general insights from their own ME-VAE model. This helps address many of the concerns I raised - for example, the reason I emphasized spatial homogeneity is because this relates to the pixel-based loss of the model they used (fluorescence distributed evenly across the cell is easier to reconstruct with this loss, which has an "averaging" property on the image, than more "spiky" fluorescence patterns such as those distributed in punctuate compartments), but adversarial losses could easily overcome this restriction.

Comment 1.1:

- Similarly, the reason I emphasized preprocessing is that it is not clear if these insights would generalize from images where the nuclei is identified and dilated versus an actual more accurate cell segmentation.

Response 1.1:

- Stain prediction methods have been shown to be successful with various segmentation methods and modalities [12,24]. Since we did not directly test multiple segmentation methods here for the reduction scope, we have added a clarification of this to the revised manuscript and suggested further research into the generalizability to multiple segmentations.

Comment 1.2:

- However, I still think that the authors need to go further in emphasizing these limitations in their writing, because there is the danger of readers still reading this paper and interpreting the insight that correlated-based selection is generally an effective strategic agnostic of methods. This is not tested, and cannot be concluded from this paper, and where I was originally misled. I appreciate how the authors have discussed the experimental limitation of their work (i.e. you do need a pilot staining where all the stains are produced first in order to be able to decide on the subset), but I think the authors should further discuss the technical limitation that what selection methods are effective are going to be dependent on the intended stain imputation method. Again, there is reason to believe the reason why a correlation-based method has done relatively well here could be because of the loss function used, and this may not be a general result over different loss functions.

Response 1.2:

- We have added a statement to the paper describing the limitation of only testing a single imputation architecture, and suggested further research into the degree to which the architecture effects the reduction methods.

Comment 1.3:

- Similarly, I still take issue with how the authors are framing their panel selection method as one that allows users to "maximize the amount of information gained" in general, as opposed to in context of a specific stain prediction method. Stains that are difficult for one method to predict may be easy for another method to predict: in other words, this is not a method that allows users to reduce stains that share information with other stains in general, but a method that reduces stains that share information with other stains *to the extent in which the empirical method used can reconstruct this information, reflecting the inductive biases and limitations of the model*

Response 1.3:

- We have clarified that the findings of this work are not a general evaluation of stain overlap or reducibility and that the findings are only with respect to the metrics used and the biases of the single imputation method tested. We acknowledge that different methods, metrics, and biases will favor different reduced panel sets, however, in this paper we are documenting the evaluation of one such imputation with a series of metrics we chose to be pertinent to evaluating the reconstruction of information.

**Comments from Reviewer #2**
Summary

The authors have greatly improved the manuscript. I only have one remaining concern regarding the train/test split.

Comment 2.1:

- The authors mention train/test split is done at the cellular image level, suggesting some cells of a patient’s image are in the train set, and some are in the test set. This is concerning as patient ID becomes a confounder. Therefore, the results of Figure 5 are not reliable. To show the network can use biologically relevant information and not data artifacts, it is necessary to split data at the patient level.

Response 2.1:

- It is correct that data from all patients is used during testing and training, meaning we cannot make claims about generalizability to new or unseen patients. We decided to do this because of the relatively small dataset size. We revised the manuscript to clearly state that generalizability to new patients is not shown in this work. Here we show the methods application and successful reconstruction across several breast cancer subtypes and pathological states. What we are showing in figure 5 is the capacity for the designed reduced panels to capture a large range of information pertinent for several cancer subtypes and to generalize across them all with a single panel. We are not making a claim about being applicable to new/unseen cancer types or patients (as we describe in our discussion). Currently we state that as a limitation which would require a pilot panel which will be used to design a new reduced panel based on it. We recommend future research into unseen patient generalizability in a larger cohort.

**Comments from Reviewer #3**
Summary

The authors have adequately responded to all my concerns.

Comment 3.1:

- The authors uploaded a new github repository: https://github.com/GelatinFrogs/PanelSelectionMethods which is a good first step in ensuring reproducibility. I should have been more specific in my original review comment, which stated:

"The authors should make their analysis code publicly available. The authors present

https://github.com/GelatinFrogs/MEVAE_Architecture, but this

repository does not contain the analysis code used in this paper from

what I could tell."

The updated repository contains the panel selection code, but no implementation instructions. The authors should also archive their github code using a service like zenodo to ensure that the citable code exists elsewhere beyond the github repository.

Response 3.1:

- We have included two more additional scripts in the github repository under the Evaluation subfolder detailing the analysis of individual reduction methods and the comparison of correlations across all methods.
